# Supplementary material for: An Agentic AI Pipeline for an Evidence-Grounded Environmental Health Public Information System: Development and Evaluation
Source: medRxiv. 2026 Oct 29:2026.10.26.26366549. Preprint. [Version 1] doi: 10.64898/2026.10.26.26366549 (PMC13734176; doi:10.64898/2026.10.26.26366549)
Supplement: Supplementary file 1 [file NIHPP2026.10.26.26366549v1-supplement-1.pdf]

Supplementary Materials

Table S1. Functional components of the agentic AI pipeline

Component	Input	Output	Purpose
Intent Parsing	User's natural language query	User intent and extracted entities (e.g., location)	Identifies the user's information need and extracts parameters required for downstream tool execution.
Location Resolution (GeoPy/Nominatim)	City, state, or place name	Geographic coordinates (latitude, longitude)	Converts user-specified locations into spatial coordinates for location-based API queries.
Weather Retrieval Tool	Geographic coordinates	Real-time weather observations and forecasts	Retrieves localized meteorological information from the NOAA API, with automatic fallback to the Synoptic Data API in the event of service interruptions.
Air Quality Retrieval Tool	Geographic coordinates	Pollutant measurements (PM2.5, PM10, O3)	Retrieves current air quality information from the AirNow API, with automatic fallback to the Synoptic Data API when data are unavailable or sensor coverage is limited.
Document Processing (Docling)	PDF guideline documents	Structured Markdown documents	Converts unstructured documents into a layout-preserving format suitable for retrieval and semantic indexing.
Document Chunking	Markdown documents	Semantically coherent text chunks	Splits documents while preserving section boundaries to improve retrieval accuracy.
Embedding	Text chunks	Dense vector	Encodes document chunks into semantic vector representations

medRxiv preprint doi: https://doi.org/10.64898/2026.10.26.26366549; this version posted October 29, 2026. The copyright holder for this preprint (which was not certified by peer review) is the author/funder, who has granted medRxiv a license to display the preprint in perpetuity.
It is made available under a CC-BY 4.0 International license .

Generation		embeddings	using the OpenAI text-embedding-3-small model.
Vector Database (ChromaDB)	Vector embeddings	Indexed semantic knowledge base	Stores embeddings and enables efficient similarity-based retrieval for Retrieval-Augmented Generation (RAG).
Health Evidence Retrieval (RAG)	User query	Relevant guideline passages	Retrieves evidence-based health information from the vector database using semantic similarity search.
Response Synthesis	Outputs from weather, air quality, and RAG tools	Evidence-grounded natural language response	Integrates information from multiple authoritative sources into a coherent, interpretable response with supporting evidence.

Table S2: Benchmark question set and required tools

id	Question type	question	Required tools
Q01	Current PM2.5 / asthma	I have asthma and I am in Sugar House right now. Is it okay to go for a walk outside?	Geocoder + Air Quality Retrieval (AirNow; Synoptic fallback) + Asthma Guideline RAG

medRxiv preprint doi: https://doi.org/10.64898/2026.10.26.26366549; this version posted October 29, 2026. The copyright holder for this preprint (which was not certified by peer review) is the author/funder, who has granted medRxiv a license to display the preprint in perpetuity.
It is made available under a CC-BY 4.0 International license .

Q02	Current PM2.5 / asthma	My child has asthma. We are near the University of Utah. Should we play outside now?	Geocoder + Air Quality Retrieval (AirNow; Synoptic fallback) + Asthma Guideline RAG
Q03	Current PM2.5 / asthma	Is the air around downtown Salt Lake City bad for asthma today?	Geocoder + Air Quality Retrieval (AirNow; Synoptic fallback) + Asthma Guideline RAG
Q04	Forecast-based air quality	I have asthma. Should I plan an outdoor run tomorrow morning in Cottonwood Heights?	Geocoder + Weather Retrieval (NOAA;

medRxiv preprint doi: https://doi.org/10.64898/2026.10.26.26366549; this version posted October 29, 2026. The copyright holder for this preprint (which was not certified by peer review) is the author/funder, who has granted medRxiv a license to display the preprint in perpetuity.
It is made available under a CC-BY 4.0 International license .

			Synoptic fallback) + Air Quality Retrieval (AirNow; Synoptic fallback) + Asthma Guideline RAG
Q05	Forecast-based air quality	Will tomorrow evening be better for asthma symptoms in Sandy compared with this afternoon?	Geocoder + Weather Retrieval (NOAA; Synoptic fallback) + Air Quality Retrieval (AirNow; Synoptic fallback) + Asthma Guideline RAG

medRxiv preprint doi: https://doi.org/10.64898/2026.10.26.26366549; this version posted October 29, 2026. The copyright holder for this preprint (which was not certified by peer review) is the author/funder, who has granted medRxiv a license to display the preprint in perpetuity.
It is made available under a CC-BY 4.0 International license .

Q06	Forecast-based air quality	Can I schedule soccer practice for kids with asthma tomorrow after school in Millcreek?	Geocoder + Weather Retrieval (NOAA; Synoptic fallback) + Air Quality Retrieval (AirNow; Synoptic fallback) + Asthma Guideline RAG
Q07	Ventilation / inversion	Is there an inversion risk in Salt Lake City this morning that could worsen asthma?	Geocoder + Weather Retrieval (NOAA; Synoptic fallback) + Air Quality Retrieval (AirNow; Synoptic

medRxiv preprint doi: https://doi.org/10.64898/2026.10.26.26366549; this version posted October 29, 2026. The copyright holder for this preprint (which was not certified by peer review) is the author/funder, who has granted medRxiv a license to display the preprint in perpetuity.
It is made available under a CC-BY 4.0 International license .

			fallback) + Asthma Guideline RAG
Q08	Ventilation / inversion	Why does my asthma feel worse in the valley on cold mornings?	Weather Retrieval (NOAA; Synoptic fallback) + Air Quality Retrieval (AirNow; Synoptic fallback) + Asthma Guideline RAG
Q09	Ventilation / inversion	Check if the air will be trapped tonight in South Jordan and whether that matters for asthma.	Geocoder + Weather Retrieval (NOAA; Synoptic fallback) + Air Quality

medRxiv preprint doi: https://doi.org/10.64898/2026.10.26.26366549; this version posted October 29, 2026. The copyright holder for this preprint (which was not certified by peer review) is the author/funder, who has granted medRxiv a license to display the preprint in perpetuity.
It is made available under a CC-BY 4.0 International license .

			Retrieval (AirNow; Synoptic fallback) + Asthma Guideline RAG
Q10	Ventilation / inversion	Is weak wind today making PM2.5 worse for people with asthma in Murray?	Geocoder + Weather Retrieval (NOAA; Synoptic fallback) + Air Quality Retrieval (AirNow; Synoptic fallback) + Asthma Guideline RAG
Q11	Gap wind / lake-breeze modifier	I live near Parleys Canyon and have asthma. Could canyon winds affect air quality this afternoon?	Geocoder + Weather Retrieval

medRxiv preprint doi: https://doi.org/10.64898/2026.10.26.26366549; this version posted October 29, 2026. The copyright holder for this preprint (which was not certified by peer review) is the author/funder, who has granted medRxiv a license to display the preprint in perpetuity.
It is made available under a CC-BY 4.0 International license .

			(NOAA; Synoptic fallback) + Air Quality Retrieval (AirNow; Synoptic fallback) + Asthma Guideline RAG
Q12	Gap wind / lake-breeze modifier	Will a lake breeze help clear the air in Salt Lake City later today for someone with asthma?	Geocoder + Weather Retrieval (NOAA; Synoptic fallback) + Air Quality Retrieval (AirNow; Synoptic fallback) + Asthma Guideline

medRxiv preprint doi: https://doi.org/10.64898/2026.10.26.26366549; this version posted October 29, 2026. The copyright holder for this preprint (which was not certified by peer review) is the author/funder, who has granted medRxiv a license to display the preprint in perpetuity.
It is made available under a CC-BY 4.0 International license .

			RAG
Q 13	Asthma-sensitive recommendation	What PM2.5 level is concerning for someone with asthma?	Asthma Guideline RAG
Q 14	Asthma-sensitive recommendation	What should I do if PM2.5 is high and my asthma is acting up?	Asthma Guideline RAG
Q 15	Asthma-sensitive recommendation	Does outdoor exercise make asthma worse when air quality is poor?	Asthma Guideline RAG
Q 16	Ambiguous location	I have asthma. Is it safe to go outside in Springfield right now?	Location clarification → Geocoder + Air Quality Retrieval (AirNow; Synoptic fallback) + Asthma Guideline RAG
Q	Ambiguous/incom	Is the air okay for my asthma here?	Location

medRxiv preprint doi: https://doi.org/10.64898/2026.10.26.26366549; this version posted October 29, 2026. The copyright holder for this preprint (which was not certified by peer review) is the author/funder, who has granted medRxiv a license to display the preprint in perpetuity.
It is made available under a CC-BY 4.0 International license .

17	plete location		clarification → Geocoder + Air Quality Retrieval (AirNow; Synoptic fallback) + Asthma Guideline RAG
Q18	Missing/conflicting data	PurpleAir sensors near me look very different from each other. What should I trust for asthma decisions?	Location clarification/Geocoder + Air Quality Retrieval (AirNow; Synoptic fallback) + Asthma Guideline RAG

medRxiv preprint doi: https://doi.org/10.64898/2026.10.26.26366549; this version posted October 29, 2026. The copyright holder for this preprint (which was not certified by peer review) is the author/funder, who has granted medRxiv a license to display the preprint in perpetuity.
It is made available under a CC-BY 4.0 International license .

Q19	Missing-data scenario	There are no nearby PM2.5 sensors for my location. Can you still advise me about asthma risk?	Geocoder + Air Quality Retrieval (AirNow; Synoptic fallback) + Asthma Guideline RAG
Q20	No-tool educational	What is PM2.5 and why can it trigger asthma?	Asthma Guideline RAG
Q21	Wildfire smoke / current asthma risk	I have an appointment at the University of Utah Pulmonology clinic today for my asthma. Are the wildfires near the university still affecting the air quality, or do I need to reschedule?	Geocoder + Air Quality Retrieval (AirNow; Synoptic fallback) + Weather Retrieval (NOAA; Synoptic

medRxiv preprint doi: https://doi.org/10.64898/2026.10.26.26366549; this version posted October 29, 2026. The copyright holder for this preprint (which was not certified by peer review) is the author/funder, who has granted medRxiv a license to display the preprint in perpetuity.
It is made available under a CC-BY 4.0 International license .

			fallback) + Asthma Guideline RAG
Q22	Wind-blown dust / asthma risk	My flight from Salt Lake City airport has been delayed by 6 hours today, and I am planning to go for a walk around the airport. Will wind raise dust from Salt Lake and exacerbate my asthma?	Geocoder + Weather Retrieval (NOAA; Synoptic fallback) + Air Quality Retrieval (AirNow; Synoptic fallback) + Asthma Guideline RAG
Q23	Current PM2.5 / outdoor exercise	I plan to go for a run in the Research Park area of the University of Utah. Is now a good time to exercise outdoors in this area?	Geocoder + Air Quality Retrieval (AirNow; Synoptic

medRxiv preprint doi: https://doi.org/10.64898/2026.10.26.26366549; this version posted October 29, 2026. The copyright holder for this preprint (which was not certified by peer review) is the author/funder, who has granted medRxiv a license to display the preprint in perpetuity.
It is made available under a CC-BY 4.0 International license .

			fallback) + Asthma Guideline RAG
Q24	Forecast-based air quality	I am based at 421 Wakara Way, Salt Lake City. I have asthma. Should I plan an outdoor run tomorrow morning?	Geocoder + Weather Retrieval (NOAA; Synoptic fallback) + Air Quality Retrieval (AirNow; Synoptic fallback) + Asthma Guideline RAG
Q25	Time-specific outdoor exercise	My location is 40.759, -111.828. When is the best time for an outdoor run?	Coordinate-to-location resolution + Weather Retrieval

medRxiv preprint doi: https://doi.org/10.64898/2026.10.26.26366549; this version posted October 29, 2026. The copyright holder for this preprint (which was not certified by peer review) is the author/funder, who has granted medRxiv a license to display the preprint in perpetuity.
It is made available under a CC-BY 4.0 International license .

			(NOAA; Synoptic fallback) + Air Quality Retrieval (AirNow; Synoptic fallback) + Asthma Guideline RAG

Table S3: Functional components of the agentic AI pipeline

Question	Tool-needed recognition	Correct required tool selection	Avoidance of irrelevant tools	Correct parameter mapping	Correct analytic module or guideline pathway	Correct incorporation of tool outputs into final answer	Total
Q01	1	1	1	1	1	1	6
Q02	1	1	1	1	1	1	6
Q03	1	1	1	1	1	1	6
Q04	1	1	1	1	1	1	6
Q05	1	1	1	1	1	1	6
Q06	1	0	1	1	0	1	4
Q07	1	0	1	1	1	1	5
Q08	0	0	1	0	0	0	1

medRxiv preprint doi: https://doi.org/10.64898/2026.10.26.26366549; this version posted October 29, 2026. The copyright holder for this preprint (which was not certified by peer review) is the author/funder, who has granted medRxiv a license to display the preprint in perpetuity.
It is made available under a CC-BY 4.0 International license .

Q09	1	1	1	1	1	1	6
Q10	1	1	1	1	1	1	6
Q11	1	0	1	1	0	1	4
Q12	1	1	1	1	1	1	6
Q13	1	1	1	1	1	1	6
Q14	1	1	1	1	1	1	6
Q15	1	1	1	1	1	1	6
Q16	1	1	1	0	1	1	5
Q17	1	1	1	1	1	1	6
Q18	1	1	1	1	0	1	5
Q19	1	0	1	1	0	1	4
Q20	1	1	1	1	1	1	6
Q21	1	1	1	1	1	1	6
Q22	1	1	1	1	1	1	6
Q23	1	1	1	1	1	1	6
Q24	1	1	1	1	1	1	6
Q25	1	1	1	1	1	1	6

medRxiv preprint doi: https://doi.org/10.64898/2026.10.26.26366549; this version posted October 29, 2026. The copyright holder for this preprint **(which was not certified by peer review)** is the author/funder, who has granted medRxiv a license to display the preprint in perpetuity.
It is made available under a CC-BY 4.0 International license .

Figure S1: Agent execution trace for Q02: asthma-sensitive outdoor activity query near the University of Utah.

{"question_id": "Q02",
"user_question": "My child has asthma. We are near the University of Utah. Should we play outside now?",
"agent_trajectory": [
"agent_node",
"tools_node",
"agent_node",
"tools_node",
"agent_node"
],
"tool_calls": [
{"tool": "air_quality",
"source": "AirNow",
"location": "Salt Lake City, UT",
"observations": {
"ozone_aqi": 31,
"pm25_aqi": 39,
"pm10_aqi": 9,
"air_quality_category": "Good"
}
},
{"tool": "rag",
"query": "asthma children outdoor play air quality guidelines",
"retrieved_sources": [
"Guarnieri2014: Outdoor air pollution and asthma",
"Tiotiu2020: Impact of Air Pollution on Asthma Outcomes"
]
}
], "final_response_summary": "Current air quality near the University of Utah was in the Good category. The agent integrated current AirNow observations with retrieved asthma evidence and advised that outdoor play was generally appropriate while monitoring the child's symptoms.",
"caption": "The agent used the AirNow air-quality retrieval tool and an asthma-focused RAG module to synthesize a context-aware recommendation for a child with asthma."
}

medRxiv preprint doi: https://doi.org/10.64898/2026.10.26.26366549; this version posted October 29, 2026. The copyright holder for this preprint (which was not certified by peer review) is the author/funder, who has granted medRxiv a license to display the preprint in perpetuity.
It is made available under a CC-BY 4.0 International license .
